# 77523 Prospective cohort study of predominantly immigrant people with chronic hepatitis B in the Baltimore-metropolitan Washington D.C. area

**DOI:** 10.1017/cts.2021.705

**Published:** 2021-03-30

**Authors:** Lydia Tang

**Affiliations:** Institute of Human Virology, University of Maryland School of Medicine, Program in Oncology, University of Maryland Marlene and Stewart Greenebaum Comprehensive Cancer Center

## Abstract

ABSTRACT IMPACT: Building a patient cohort to support clinical-translational research in chronic hepatitis B OBJECTIVES/GOALS: The overall objective of my KL2 project is to delineate the effect of HIV coinfection, and CHB disease stage and hepatitis B (HBV) viremia on the ability of toll-like receptor 8 agonism to restore HBV-specific immune cell function. This abstract describes the characteristics of the cohort from which research blood samples for my KL2 are collected. METHODS/STUDY POPULATION: HOPE is a prospective cohort study enrolling people with CHB including HIV/CHB coinfection, and resolved CHB. Participants are enrolled at primary care clinics in Maryland, Washington D.C., and Virginia. Standard-of-care antiviral treatment with tenofovir alafenamide (TAF) is prescribed through the study if indicated. Patients receiving TAF from the study are either starting treatment, or switching to TAF from another antiviral medication. If receiving TAF, participants are seen every 3-6 months for medication refills, clinical and research blood draws, and adverse event evaluations. Liver fibrosis is measured by FibroScan and a minority undergo liver biopsy. RESULTS/ANTICIPATED RESULTS: So far, 204 people have been enrolled, 177 with CHB (23 HIV/CHB coinfection), and 22 with resolved HBV infection. To date, 45 patients who were viremic at baseline and initiated on TAF have been enrolled. CHB predominantly affects Asian and African immigrants in the U.S, and the majority (77%) of HOPE participants are immigrants from these countries. The majority are male (70%), mean age 51 years (SD ±14). So far, 86 people with CHB monoinfection have been prescribed TAF on-study. Thirty-five percent of these had significant (>F2) liver fibrosis at baseline and half had elevated ALT (mean 47.5, SD ±55 IU/ml). Forty-three percent switched to TAF from another oral antiviral. Most switched due to lack of coverage by health insurance. DISCUSSION/SIGNIFICANCE OF FINDINGS: HOPE is a CHB cohort dedicated to collecting research samples and providing antiviral treatment. It is the foundation for the CHB translational research program at the University of Maryland School of Medicine. The availability of paired viremic and virally suppressed, HIV/CHB, and resolved HBV research samples are strengths of HOPE.

